# Persistent and de novo postpartum hypertension: a scoping review of pathophysiology, evaluation, and management

**DOI:** 10.1038/s44325-026-00120-x

**Published:** 2026-05-04

**Authors:** Mansi Garneni, Alice Huang, Ifeoma Obionu, Zainab Mahmoud

**Affiliations:** 1 Washington University School of Medicine, Washington University in Saint Louis, Saint Louis, MO USA; 2 Cardiovascular Division, Department of Medicine, University of Minnesota Medical School, Minneapolis, MN USA; 3 Division of Cardiology, Department of Medicine, Washington University in Saint Louis, Saint Louis, MO USA; 4 Department of Obstetrics and Gynecology, Washington University in Saint Louis, Saint Louis, MO USA; 5 School of Public Health, Washington University in Saint Louis, Saint Louis, MO USA

**Keywords:** Cardiology, Diseases, Health care, Medical research

## Abstract

Postpartum hypertension is high blood pressure following delivery that may occur as persistent or de novo postpartum hypertension. This scoping review summarizes current evidence on the epidemiology, risk factors, evaluation, and management of postpartum hypertension and highlights areas for further investigation. Studies on persistent or de novo hypertension in the 12-month postpartum period were eligible for inclusion. Literature searches were performed in MEDLINE, Embase, and Scopus. Of 2132 studies screened, 116 met the inclusion criteria. Postpartum hypertension epidemiologic data are limited, in part due to the absence of a standardized definition. Distinct risk factors for persistent versus de novo disease suggest differing underlying pathophysiologic mechanisms, yet trials of postpartum antihypertensive therapies have not identified a clear first-line agent. While telemedicine interventions have demonstrated improvements in postpartum hypertension outcomes, standardization of diagnostic and treatment thresholds and expanded research into mechanisms and therapeutics for persistent and de novo disease are needed.

## Introduction

Postpartum hypertension, defined as elevated blood pressure following delivery, is an increasingly recognized contributor to maternal morbidity in the early postpartum period^1–3^. Approximately 10% of patients without preexisting hypertension develop elevated blood pressure within one year after pregnancy^4^. Postpartum hypertension may present as either persistent hypertension or develop de novo following delivery, with the latter affecting approximately 2% of pregnancies within the first 6 weeks postpartum^5^. Although the early postpartum period is a time of significant physiological transition, it remains underrecognized in both clinical care and research. During this vulnerable period, blood pressure can fluctuate substantially, placing patients at increased risk for hypertensive complications, including severe hypertension, stroke, and heart failure^4,6,7^. Despite these risks, the natural history, pathophysiology, and optimal management strategies for postpartum hypertension remain poorly defined.

Much of the existing literature and clinical focus has centered on the antenatal period, particularly on hypertensive disorders of pregnancy (HDP), which affect approximately 10% of pregnancies globally and remain a leading cause of maternal morbidity and mortality^5^. HDP include chronic hypertension, gestational hypertension, preeclampsia spectrum including eclampsia and HELLP syndrome, and chronic hypertension with superimposed preeclampsia^4,8^. However, postpartum hypertension is not simply an extension of HDP. Increasing evidence suggests that the postpartum phase represents a distinct period of hypertensive risk, with both persistent and de novo hypertension contributing to adverse outcomes^4^. A comprehensive understanding of postpartum hypertension, from its epidemiology, risk factors, clinical trajectories, to the treatment approaches, is essential to close existing knowledge gaps and inform strategies to improve maternal cardiovascular outcomes.

The evidence around postpartum hypertension has evolved significantly over the past decade. Updates in diagnostic criteria in non-pregnant adults, including the adoption of lower blood pressure thresholds (<130/80 mm Hg), have expanded recognition of postpartum hypertension, particularly de novo cases that were previously underdiagnosed. Additionally, emerging evidence underscores the importance of long-term cardiovascular monitoring, as postpartum hypertension is associated with increased risks of recurrent HDP, stroke, heart failure, and other cardiovascular complications^9^. Furthermore, advancements in remote blood pressure monitoring and pharmacological therapies necessitate the need for an updated synthesis of management strategies. Given these developments, the objective of this scoping review was to synthesize the existing literature and provide updated evidence on persistent and de novo postpartum hypertension, with a focus on epidemiology, pathophysiology, evaluation, and management; to map key concepts; identify gaps in evidence; and inform future research and clinical practice in this understudied area of maternal cardiovascular health. Unlike prior reviews, this is the first scoping review to comprehensively examine both persistent and de novo postpartum hypertension, integrating evidence across epidemiology, pathophysiology, evaluation, and management^1,2,5,10^.

Current management guidelines for postpartum hypertension are largely extrapolated from studies on hypertensive disorders during pregnancy or on primary hypertension, with limited attention to the unique pathophysiology and clinical course of postpartum hypertension. Given the various terminology used across studies, we have included a table of definitions for clarity (Table 1). This review focuses on subacute to chronic elevations in blood pressure during the postpartum period (i.e., persistent postpartum hypertension and de novo postpartum hypertension) rather than acute hypertensive emergencies following delivery.

**Table 1:** Definitions of hypertension terms related to pregnancy and the postpartum period

Terms	Definitions
Chronic hypertension	Elevated blood pressure was diagnosed before 20 weeks of pregnancy or prior to pregnancy.
Gestational hypertension	Elevated blood pressure (≥140/90) developing after 20 weeks of pregnancy without signs of preeclampsia.
Postpartum hypertension	Elevated blood pressure occurs after delivery. Includes all types of hypertension during the postpartum period.
Persistent postpartum hypertension	Hypertension that develops during pregnancy (gestational hypertension/preeclampsia) but continues postpartum.
De novo postpartum hypertension	New onset of hypertension that arises postpartum following a normotensive pregnancy.

## Results

**Fig. 1:**
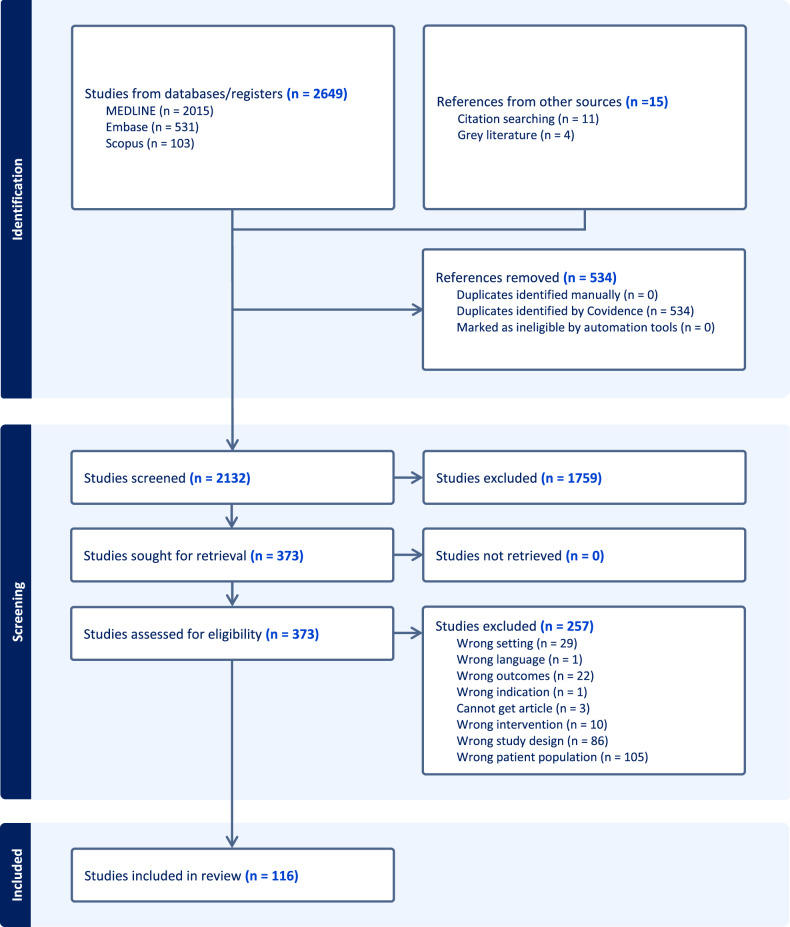
PRISMA flow diagram for the scoping review of persistent and de novo postpartum hypertension. Flow diagram illustrating the identification, screening, eligibility assessment, and inclusion of studies for the scoping review on postpartum hypertension. A total of 2649 records were identified from databases, and 15 additional records from other sources. After removal of duplicates and screening, 116 studies met the inclusion criteria and were included in the final review

A total of 2293 citations were imported into Covidence. After automated and manual deduplication, 1897 unique citations remained for screening. The second search yielded an additional 356 records, of which 138 were duplicates. In total, 218 unique new records were screened following the updated search. An additional 15 studies were added from gray literature and through citation searching. During the initial screening of titles and abstracts, 1759 citations were excluded due to irrelevance to the study objectives. 373 records underwent full-text review. A further 257 were excluded for reasons highlighted in Fig. 1. 116 studies met the inclusion criteria.

### Epidemiology of persistent and de novo postpartum hypertension

The prevalence of postpartum hypertension varies across populations and clinical contexts. A prospective study of 115 women with preeclampsia in Switzerland found that 50% had persistent hypertension at both 6 and 12 weeks postpartum^11^. In a systematic review, Giorgione et al. reported a 28% incidence of persistent hypertension (468/1646 patients) following preeclampsia and a 9% incidence of de novo hypertension (584/6395 patients) at 2 years postpartum^12^. Also, a study of 2465 women with normotensive pregnancies at a large safety-net hospital in the United States observed that 12.1% developed new-onset hypertension within 1 year of delivery^4^. Approximately 22% of those cases were diagnosed after 6 weeks postpartum. Similarly, Smith et al.’s study of 1366 participants found that 22% of women with otherwise uncomplicated pregnancies in their study had elevated blood pressure at 1 year postpartum^13^. In a cohort of 7730 patients, Sinnott et al. showed 8.2% of patients developed de novo postpartum hypertension between 6 weeks and 6 months postpartum^14^. In a Japanese study of 18,295 normotensive women, the incidence of de novo postpartum hypertension was only 1.2%, demonstrating the variability that exists across a wide range of clinical contexts^15^. Currently, the authors are not aware of any available epidemiologic data that encapsulates the worldwide incidence and prevalence of postpartum hypertension.

### Pathophysiologic mechanisms of persistent postpartum hypertension

Multiple studies have explored potential mechanisms contributing to persistent hypertension in the postpartum period, particularly among women with prior hypertensive disorders of pregnancy. The evidence was primarily observational. Zhou et al. reported that elevated maternal lipoprotein-associated phospholipase A2 levels were associated with postpartum hypertension following preeclampsia (OR 1.134, 95% CI 1.086–1.185)^16^. Barr et al. implicated persistent microvascular dysfunction and arteriolar vasoconstriction as underlying contributors^17^. While placental angiogenic biomarkers such as soluble fms-like tyrosine kinase-1 (sFlt-1) and placental growth factor (PlGF) have been studied extensively in preeclampsia, their predictive utility for postpartum hypertension remains unclear. Neuman et al. found no significant association between these markers and postpartum blood pressure at 1 year (OR 0.9, 95% CI 0.2–4.4)^18^. However, a small pilot study by Hamza et al. suggested that elevated postpartum PlGF levels may correlate with higher systolic blood pressure^19^. Also, Biwer et al. suggest that exposure to sFlt-1 in pregnancy increases salt-sensitivity of postpartum blood pressure^20^.

### Pathophysiologic mechanisms for de novo postpartum hypertension

Emerging evidence on the pathogenesis of de novo postpartum hypertension is mainly observational and suggests residual vascular and endothelial abnormalities from pregnancy may play a role. Proposed mechanisms include delayed resolution of pregnancy-induced vascular adaptations, sustained antiangiogenic imbalance (e.g., elevated sFlt-1 and reduced PlGF), endothelial dysfunction, autonomic dysregulation, inflammatory processes, and aberrant fluid and sodium homeostasis^21–23^. Additionally, Kim et al. identified differential placental CpG methylation patterns in women who developed new-onset postpartum preeclampsia, suggesting a possible role for epigenetic modifications^24^.

### Risk and protective factors for postpartum hypertension

Several risk and protective factors for developing postpartum hypertension have been identified. With regards to the risk factors for postpartum hypertension, these can be further divided into risk factors for persistent postpartum hypertension and de novo postpartum hypertension, although significant overlap exists. Table 2 details the various risk factors for postpartum hypertension.

**Table 2:** Risk factors for persistent and de novo postpartum hypertension

System category	Persistent postpartum hypertension	De novo postpartum hypertension
Cardiovascular	Early onset of hypertension with end-organ dysfunction^28^ Elevated blood pressure in the immediate postpartum period^97^ Elevated B-type natriuretic peptide^98^ Higher left ventricular wall thickness^93^ First-degree family history of cardiovascular disease or chronic hypertension^99^	Elevated blood pressure before or at delivery^100^ First-degree family history of cardiovascular disease or chronic hypertension^99^
Metabolic	Pre-pregnancy BMI^28,101^ Metabolic syndrome^102^ High triglycerides in early pregnancy^103^ Gestational diabetes^25^ Excessive pregnancy weight gain^25^	Pre-pregnancy BMI^100^ High triglycerides in early pregnancy^103^ Maternal obesity^104,105^
Obstetric	High gravidity^106^ Early-onset pre-eclampsia^106,107^ Duration of preeclampsia^108^	Multifetal gestations^105^ Assisted reproductive technology^100^ Cesarean section^100,104,105,109^ History of hypertensive disorders of pregnancy in previous pregnancies^110^
Other	Age over 35^111^ Elevated creatinine at admission^112^ History of smoking^28^ Maternal HIV infection^113^ Systemic lupus erythematosis^28^ Antiphospholipid antibody syndrome^28^ Reduced eGFR at delivery^44^ Worse sleep quality^114^	Age over 35^4^ Elevated creatinine^115^ Current or former smoker^4,110^ Chronic nephritis^100^ Hypothyroidism^100^ Black patients^104,116^ Elevated aspartate aminotransferase and alanine aminotransferase^22^

Although less characterized, several protective factors for the development of postpartum hypertension have been identified. Lactation, nulliparity status, vaginal delivery, nonsmoking status, avoidance of excessive gestational weight gain, and antenatal low-dose aspirin have been shown to be protective against postpartum hypertension^4,25–30^. In Countouris et al., lactating for at least 6 months was associated with lower postpartum systolic (*β* = −16.1 mm Hg, 95% CI −27.7 to −4.5) and diastolic (*β* = −16.9 mm Hg, 95% CI −27.8 to −6.0) blood pressures in women with gestational hypertension, but no association was noted for preeclamptic or normotensive women^26^. Christenson et al. reported that antenatal administration of low-dose aspirin was associated with a reduced incidence of postpartum hypertension, as well as fewer hospital readmissions at 6 weeks postpartum, in patients with preeclampsia^27^. Additionally, a possible protective factor that requires further investigation is calcium supplementation during pregnancy. Gomes et al. suggest that calcium supplementation can reduce the risk of hypertensive disorders of pregnancy, which may indirectly lower the risk of postpartum hypertension, as HDP is a risk factor for postpartum hypertension^31^.

### Evaluation and management of postpartum hypertension

The American College of Cardiology/American Heart Association (ACC/AHA) 2025 guidelines set the blood pressure goal of <130/80 mm Hg for the general population^32^. While the American College of Obstetricians and Gynecologists (ACOG) defines gestational hypertension as blood pressure of 140/90 or more on two occasions at least 4 hours apart after twenty weeks of gestation in a woman with a previously normal blood pressure^33^, currently there is no consensus on the blood pressure thresholds at which postpartum hypertension is diagnosed or treated^8,34^. This inconsistency leads to variability in clinical practice in the postpartum period and makes it challenging to accurately assess the incidence and prevalence of postpartum hypertension^35^.

Recent evidence suggests that the threshold to treat postpartum hypertension may be set too high, potentially excluding patients who should benefit from timely risk-reducing interventions^13,36^. Further, there is data showing that clinicians should aim for tighter blood pressure control in the postpartum period^37^. Smith et al. demonstrated that 22% of postpartum patients in their cohort had hypertension at 1 year postpartum per the new ACC/AHA guidelines (>130/80 mm Hg), but less than 1% of the patients would have been diagnosed with hypertension per the old criteria (>140/90 mm Hg)^13^. This lower standard appears safe to target: Sarma et al., a pilot study of 62 patients, demonstrated that titrating blood pressure medication in line with ACC/AHA guidelines, to achieve a goal BP of <130 mmHg, resulted in optimal BP control at 6 weeks postpartum, with no patients having hypotensive events^38^.

Tighter blood pressure control reduces emergency department visits and hospital readmissions compared to standard thresholds^37,39^. In Rosenfeld et al., a multicenter study of 705 women with a diagnosis of hypertensive disorder, targeting a tighter blood pressure goal (<130/80 mm Hg) compared to standard control (<150/100 mm Hg) led to a 68% reduction of postpartum emergency department utilization for hypertension and a modest reduction in systolic and diastolic blood pressure at 6-week follow-up^37^. By contrast, Aderibigbe et al. showed no difference in readmission or persistence of hypertension beyond 14 days with postpartum initiation of antihypertensive therapy at <140/90 versus <150/95 mm Hg in patients with HDP^40^. Notably, the tighter control group had higher BMI at delivery (37.1 ± 9.4 vs. 34.9 ± 8.1; *p* = 0.04), suggesting that pregnancy weight gain and BMI affect postpartum cardiovascular risk^40^.

In terms of timing of postpartum follow-up, ACOG recommends blood pressure follow-up within 7–10 days postpartum, or within 3 days for women with severe hypertension^41^. Other experts have recommended follow-up for blood pressure monitoring, among other postpartum care monitoring at 3–5 days after delivery, when blood pressure typically peaks^41–43^. Studies support monitoring for symptoms within the week after delivery and having follow-up past the standard 6-week postpartum visit to account for fluctuations in postpartum blood pressure and diagnose de novo postpartum hypertension^4,11,43–48^. Ackerman-Banks et al. reported that patients with HDP have a 4-fold increased risk of developing persistent postpartum hypertension within 1 year postpartum compared to non-HDP patients (aOR 4.60, 95% CI 1.65–12.81), suggesting that 6-week follow-up is insufficient, especially for high-risk patients^48^.

There is limited data on a standardized workup for postpartum hypertension when it is first diagnosed. Work-up for secondary causes of hypertension during the postpartum period should be initiated if not done already and suspicion is high; for example, in patients with maternal age under 35 years, severe or resistant hypertension, strong family history of renal disease, laboratory abnormalities such as hypokalemia, albuminuria, elevated creatinine, or clinical features and symptoms suggestive of obstructive sleep apnea^2,49^. Ghuman et al. suggest the work-up for a patient with postpartum hypertension should include a complete blood count, comprehensive metabolic panel, urinalysis, and renal and thyroid function testing to check for secondary causes^50^. These laboratory tests would aid in capturing such secondary causes of postpartum hypertension as chronic kidney disease, hypothyroidism or hyperthyroidism, new-onset postpartum preeclampsia, to name a few. Lewey et al. noted that a sleep study should be recommended to assess for obstructive sleep apnea in individuals with obstructive sleep apnea during pregnancy, with symptoms like sleep disturbances, daytime sleepiness, snoring, and/or obesity^49^. Additionally, if clinical concern exists for primary hyperaldosteronism, measurement of plasma renin and aldosterone levels is advised. However, it is noted that these values may have limited diagnostic utility in the immediate postpartum period, as renin and aldosterone levels remain suppressed during pregnancy and typically normalize within 6 weeks postpartum^49^.

### Pharmacologic interventions

Although several antihypertensive agents have demonstrated efficacy in the management of postpartum hypertension, current guidelines do not specify a preferred first-line therapy. Commonly used agents include labetalol, nifedipine, amlodipine, and enalapril. Table 3 outlines the various randomized controlled trials of oral postpartum antihypertensives and diuretics. These studies focus on outcomes of blood pressure control, health care utilization, and side effects. Across randomized trials, there were no significant differences in primary or secondary outcomes, namely blood pressure control, need for additional antihypertensive medications, length of stay, rapidity of achieving sustained blood pressure control, readmission for blood pressure, or patient-reported side effects, among the studied antihypertensives in postpartum hypertension^51–61^.

**Table 3:** Summary of relevant randomized controlled trials of oral postpartum antihypertensives and diuretics

Study (author, *N*)	Antihypertensive agent	Primary outcome and findings	Secondary outcomes
Ascarelli^51^ *N* = 264	Furosemide 20 mg daily × 5 days vs. Placebo	In patients with severe preeclampsia (*n* = 70), the furosemide group had lower SBP on postpartum day 2 (142 ± 13 mmHg vs. 153 ± 19 mmHg, *p* < 0.004); no benefit in mild or superimposed preeclampsia.	Furosemide did not affect length of hospitalization or frequency of delayed postpartum complications. Furosemide group required less antihypertensive therapy at discharge (6% vs. 26%, *p* = 0.045).
Sharma^52^ *N* = 50	Labetalol vs. Nifedipine ER	Time to sustained BP control similar (37.6 vs. 38.2 h, *p* = 0.51).	Labetalol more effective at initial dose (76% vs. 46%, p = 0.04); side effects more common with nifedipine (48% vs. 20%, *p* = 0.04).
Viteri^53^ *N* = 118	Torsemide vs. Placebo	Persistent HTN: 44% in torsemide vs. 58% in placebo (RR 0.76, 95% CI 0.5–1.1).	No differences in HTN at 7–10 days or 6 weeks, severe HTN, LOS, readmission, or adverse events.
Ainuddin^54^ *N* = 124	Labetalol vs. oral Nifedipine	Faster BP control with nifedipine (30.4 vs. 35.6 h, *p* = 0.04).	Similar LOS and need for additional meds; side effects more frequent with nifedipine.
Perdigao^55^ *N* = 384	Furosemide 20 mg daily × 5 days vs. Placebo	60% reduction in persistent HTN at 7 days (adjusted for cesarean); faster BP resolution in non-severe disease.	No difference in readmissions or need for additional antihypertensives.
Bartal^56^ *N* = 70	HCTZ + Lisinopril vs. Nifedipine	Stage 2 HTN at day 7–10 or readmission: 27% (HCTZ + Lisinopril) vs. 43% (Nifedipine)	No differences between groups in severe maternal morbidity (ICU admission, HELLP syndrome, eclampsia, stroke, cardiomyopathy, or maternal death), intravenous meds, LOS, clinic BP, adherence, or adverse events.
Gupta^57^ (HIPPO study) *N* = 130	Labetalol vs. Amlodipine	Amlodipine achieved BP control faster (mean difference 7.2 h; 95% CI 1.4 to 12.9, *p* = 0.011).	Fewer severe HTN episodes with amlodipine; more discharge antihypertensive use in amlodipine group.
Yoselevsky^58^ *N* = 94	Enalapril 10 mg vs. Nifedipine ER 30 mg daily	No significant difference in composite of LOS, unplanned visits, or readmissions.	More second agents added in enalapril group; more patients on therapy at 2 weeks in nifedipine group.
Emeruwa^59^ (LAPP trial) *N* = 82	Furosemide 20 mg daily × 5 days vs. Placebo	No difference in MAP averaged over 24 h before discharge or 24 h before antihypertensive therapy initiation	No difference in de novo hypertension, readmission, morbidity, breastfeeding, or neonatal outcomes.
Pratt^60^ *N* = 175	Amlodipine vs. Nifedipine ER	Amlodipine non-inferior to nifedipine ER for LOS	Similar side effects, readmission rates, breastfeeding outcomes and use of additional antihypertensives; hypotension and tachycardia are less common with amlodipine. Amlodipine was significantly less likely to be discontinued due to side effects.
Marques^61^ *N* = 172	Continue Methyldopa vs. Switch to Captopril	No difference in BP control, defined as the ability to maintain BP < 140/90 mmHg at >50% of postpartum measurements, in the first 48 h following delivery with continuing methyldopa vs. switching to captopril (92% vs. 95.2%, OR 0.58, 95% CI 0.15 to 1.99, *p* = 0.397)	No significant differences found in side effects, postpartum depression, hypertensive peaks, time to BP control, additional medications, or maternal complications

*ER* Extended release, *HCTZ* Hydrochlorothiazide, *HTN* Hypertension, *ICU* Intensive care unit, *LOS* Length of stay, *MAP* Mean arterial pressure, *SBP* Systolic blood pressure.

Overall, Irfan et al. performed a systematic review and meta-analysis of patients with persistent and de novo postpartum hypertension with a follow-up of 6 months that showed that all traditional antihypertensives were effective at managing postpartum hypertension up to 6 months^62^. Patients on ACE inhibitors or ARBs required additional antihypertensives compared to other drugs (RR 2.09, 95% CI 1.07–4.07, *p* = 0.03)^62^. Scudo et al.’s narrative review also found that one medication has not been shown to be more effective than another^63^.

Multiple randomized controlled trials and systematic reviews have shown mixed results on the efficacy of short course loop diuretics on postpartum blood pressure^51,55,59,64–66^. Perdigao et al. conducted a randomized controlled trial among women with hypertensive disorders of pregnancy and found that a 5-day course of furosemide, compared to placebo, significantly reduced the risk of persistent elevation of blood pressure at 7 days postpartum (adjusted RR 0.4, 95% CI 0.2–0.81)^55^. In the LAsix for the Prevention of de novo Postpartum hypertension (LAPP) trial, 82 participants at high risk for developing de novo postpartum hypertension were randomized to either receive a 5-day course of furosemide or placebo and were followed for 6 weeks postpartum^59^. There was no significant difference in mean arterial pressure during the 24 hours before discharge (absolute difference 2.1 mm Hg, 95% CI −1.2 to 5.3), nor in rates of de novo postpartum hypertension (8 participants developed hypertension, 7 of whom were in the furosemide group).

Even in the instances where differences between antihypertensives were observed, no single agent demonstrated clear superiority. In the Hypertension In Pregnancy & Postpartum Oral-antihypertensive therapy (HIPPO) study (2023), oral amlodipine did achieve sustained blood pressure control more rapidly than oral labetalol and did result in fewer severe hypertensive episodes, but more women in the amlodipine group required ongoing antihypertensive therapy at discharge^57^.

### Non-pharmacologic interventions

Several studies have explored the use of telemedicine, including remote health monitoring, as an intervention for postpartum hypertension. One of the earliest studies in this space was a randomized clinical trial comparing standard office-based follow-up with text-based remote monitoring in the management of postpartum hypertension^67^. 206 postpartum women with pregnancy-related hypertension diagnosed during their delivery admission were randomized to two study arms: 2 weeks of text-based surveillance using a home blood pressure cuff and a previously tested automated platform, or usual care blood pressure check at their prenatal clinic 4–6 days following discharge. The study found that there was an increase in a single blood pressure obtained in the texting group in the first 10 days postpartum compared to the office group (OR 58.2, 95% CI 16.2–208.1)^67^. Of note, the large OR of 58.2 reflects greater engagement and increased ascertainment of blood pressure measurements in the texting group, rather than an increase in incidence of hypertension. Similarly, the self-management of postnatal hypertension (SNAP-HT) trial (2018) demonstrated that postpartum self-management was feasible amongst their 186 participants, where they compared usual care to daily home BP monitoring and automated medication reduction via telemonitoring. Compared to the control group, the intervention group had improved blood pressure control at 6 weeks (systolic −5.2 mm Hg, 95% CI −9.3 to −1.2; diastolic −5.8 mm Hg, 95% CI −9.1 to −2.5) and more sustained diastolic blood pressure reduction (−4.5 mm Hg, 95% CI −8.1 to −0.8) at 6 months^68^. Altogether, various trials highlight the utility of telemedicine and self-management in postpartum hypertension monitoring^69,70^.

Long-term follow-up rates for postpartum hypertension are low, especially among Black and publicly insured patients^71,72^. The Khosla et al. retrospective cohort study documented the transition to telehealth with audio-based visits from standard in-clinic visits for a cohort of 473 postpartum hypertension patients^73^. The study found that this transition improved attendance at postpartum hypertension visits among non-Hispanic Black patients, highlighting the role that telemedicine can play in decreasing racial disparities in follow-up rates^73^. While remote monitoring can help alleviate racial disparities in attendance compared to traditional clinic visits, disparities still persist^74,75^.

Integrated, structured models of postpartum care can also improve attendance. Amro et al. demonstrated that combining maternal visits with well-child visits at intervals of 2 days, 2 weeks, and 2 months led to decreased time to readmission for persistent and de novo postpartum hypertensive disease^76^. Celi et al. described a postpartum clinic for HDP patients that provided a structured transition to postpartum follow-up care, allowing this patient population to see a nutritionist and improving provision of home blood pressure monitors (56.8-93.8%, p < 0.0001)^77^. In a multifaceted initiative described by Suresh et al., a dedicated nurse educator along with patient and provider education in inpatient and outpatient settings improved attendance and decreased BP readings above 140/90 mm Hg at the first visit^78^.

## Discussion

The lack of standardized diagnostic criteria for postpartum hypertension, especially inconsistent blood pressure thresholds, remains a major limitation that hinders comparability and surveillance across regions^79^. The most recent ACOG guidelines do not contain explicit postpartum blood pressure thresholds^41^. Similarly, while the recent ACC/AHA 2025 guidelines have a specified blood pressure treatment threshold for hypertension, it does not contain a diagnostic threshold for the diagnosis of postpartum hypertension^32^. This lack of clarity creates uncertainty about whether postpartum patients should be managed according to pregnancy-specific or nonpregnant adult thresholds, leading to variability in clinical practice. Additionally, current understanding of its epidemiology is largely derived from cohort studies of varying design, sample size and population characteristics, which limits comparability across studies. Establishing unified definitions and treatment thresholds is essential to standardize care, particularly as emerging evidence suggests that tighter blood pressure control in the postpartum period may reduce cardiovascular risk^37^.

Choice of antihypertensives for postpartum hypertension should be made on an individualized basis, with careful consideration for a patient’s comorbidities, kidney function, ability to tolerate the side effect profile, and plans for future pregnancy. Postpartum individuals require certain careful medication considerations that may not apply to the general population, such as the need for the medication to be safe for use in pregnancy and lactation.

Several head-to-head trials have compared individual blood pressure medications. However, there is a need to expand randomized controlled trials to evaluate combination regimens, as many postpartum patients require more than one blood pressure medication. For example, the Bartal et al. pilot study found that women receiving hydrochlorothiazide plus lisinopril had lower rates of stage 2 hypertension at 7–10 days postpartum or at readmission compared with those treated with nifedipine alone (27% vs. 43%)^56^.

Identifying modifiable risk and protective factors provides an opportunity to reduce patient risk in the antenatal period. Clinicians could identify patients with a higher risk for postpartum hypertension, focus on risk factors such as smoking status, weight management, and optimize treatments for chronic conditions such as HIV, hypothyroidism, and lupus nephritis to decrease the risk of de novo and persistent postpartum hypertension in pregnant patients prior to delivery or individuals planning for pregnancy. These findings support early risk stratification and medical optimization during the antenatal period.

Beyond risk reduction, patient counseling may also incorporate identified protective factors such as lactation that support better postpartum blood pressure control, particularly for high-risk patients^26,29^. This data suggests high-risk patients could benefit from interprofessional team members like lactation nurses to improve confidence and adherence with breastfeeding. Counseling on protective behaviors can complement risk reduction to support blood pressure management.

To ensure that any blood pressure elevations occurring beyond the standard postpartum follow-up period are identified and managed appropriately, patients should be transitioned to a primary care provider after their 6-week postpartum visit. No guidelines exist on how this transition should be conducted, so it should be done on an individualized basis based on patient needs, risk factors, availability of resources, and access to care. Celi et al. described a postpartum transition clinic for patients with HDP, staffed by primary care providers, that served as a bridge from obstetric care to primary care, with 79.5% of patients with in-system primary care providers attending their scheduled primary care visits. This clinic is one example of how integrated clinics can facilitate transition of care from obstetrics to primary care in the postpartum period, especially for high-risk patients.

Notably, none of the randomized controlled trials included in this review reported outcomes separately for de novo postpartum hypertension and persistent postpartum hypertension^68,80^. Further studies should examine whether different antihypertensives and diuretic therapies have different effects across subgroups. Although the etiologies of persistent and de novo postpartum hypertension remain unclear, the presence of distinct risk factors suggests they may have different underlying mechanisms. While their clinical outcomes may be similar^81^, this distinction highlights the need for further research into their pathophysiology and long-term consequences as well. Additionally, the long-term impact of different antihypertensive agents on postpartum hypertension warrants further investigation, especially since hypertension can develop several years after HDP^82,83^. Except for a few studies such as the POP-HT (2024) and SNAP-HT (2018) trials, most randomized controlled trials have follow-up durations of 6 weeks or less, often limited to the first few days postpartum^68,70^. Longer-duration trials are needed to characterize long-term blood pressure trajectories and evaluate sustained treatment effects, which would better inform medication selection and chronic disease prevention strategies in this population.

Telemedicine has transformed medical practice, including in postpartum hypertension, by providing wider access to care and timely follow-up for patients in the postpartum hypertension period^84–88^. Black patients, who face a disproportionately higher risk of HDP and postpartum complications, historically have lower rates of postpartum follow-up than White patients. Evidence suggests that experiencing structural and interpersonal racism is associated with elevated postpartum blood pressures in Black, Hispanic, and Asian patients^89^. In fact, racial disparities in postpartum hypertension persist for Black patients compared to White patients when the neighborhood area deprivation index is considered^90^. Khosla et al. demonstrated that telemedicine can help decrease racial disparities among the postpartum hypertension population by improving visit attendance, but Patel et al. showed that racial disparities in attendance still exist in remote BP monitoring models^73,75^. Telemedicine also benefits patients with transportation barriers or those living in areas with limited healthcare access, bridging critical gaps in continuity of care. However, inequalities persist within telehealth models. For example, patients may not have access to reliable internet, phone service, or doctors who can oversee the remote monitoring data, highlighting the need for infrastructure and policy support. While remote blood pressure monitoring and telehealth offer promising strategies to improve access to care in both urban and rural settings, increased advocacy is needed to expand acceptance, availability, and insurance coverage for these services.

In recent years, growing evidence has supported text message-based management and remote blood pressure monitoring in postpartum hypertension, driven by the widespread adoption of remote care models. As a result, the AHA released a 2024 scientific statement recognizing that home blood pressure monitoring and telemedicine programs improve blood pressure monitoring rates, increase postpartum obstetric visits, and reduce emergency department visits and readmissions for hypertension^49^. From a public health perspective, scaling systems-level telemedicine initiatives represents a key opportunity to advance equity and improve outcomes in the postpartum hypertension population.

Recent findings show that hypertensive disorders of pregnancy result in adverse cardiac remodeling in the postpartum period and improved postpartum blood pressure control can undo some of those effects^80,91–93^. Because pregnancy occurs in relatively young individuals with lower baseline cardiovascular risk, the postpartum period presents a unique opportunity to alter the lifetime trajectory of cardiovascular disease. In its 2024 scientific statement, the AHA highlighted labetalol, nifedipine, amlodipine, and enalapril as commonly used postpartum antihypertensives but also noted that no randomized clinical trials have evaluated the effects of postpartum interventions on long-term maternal cardiovascular disease outcomes^49^. Postpartum hypertension is significant beyond obstetrics because it reflects early vascular dysfunction that could accelerate the development of chronic hypertension and heart failure and identifies a high-risk population who may be overlooked by traditional methods of cardiovascular risk stratification. Future research could explore the impact of postpartum hypertension on long-term cardiovascular outcomes.

This scoping review’s strengths are its clinical relevance and comprehensive scope. Postpartum hypertension is an emerging area of research and clinical focus in cardio-obstetrics. Thus, there have been many studies on this topic recently. This review synthesizes the most up-to-date evidence on postpartum hypertension, focusing on key clinical takeaways for providers about risk and protective factors, evaluation, and management. Additionally, by mapping current evidence, this scoping review also highlights areas for further research.

This scoping review has several limitations. First, inconsistency across the literature regarding diagnostic criteria for postpartum hypertension and length of follow-up makes it challenging to synthesize conclusions across studies. Data on incidence, prevalence, and risk and protective factors are context-dependent, further complicating the task of creating generalizable results. Lastly, most studies have focused on the early postpartum period, typically within 6 weeks of delivery, with limited data on the long-term blood pressure trajectories and subsequent cardiovascular outcomes. Standardized, longitudinal research is needed to better understand optimal treatment strategies, preventive interventions, and long-term outcomes.

In conclusion, this review synthesizes current knowledge on the etiologies, risk and protective factors, evaluation, and management of persistent and de novo postpartum hypertension. Treatment should be individualized to the clinical context and patient preferences, taking into account continuity in this population that may become pregnant again. Long-term impacts of various antihypertensive strategies as well as other opportunities to improve outcomes like modifiable risk factors should be further studied. Future research should also clarify the pathophysiologic mechanisms of persistent versus de novo postpartum hypertension and evaluate the long-term cardiovascular outcomes of each one. Another gap is the threshold at which postpartum hypertension should be treated. Evidence suggests that tighter blood pressure control (<130/80 mm Hg) is associated with reduced morbidity, but unclear guidelines cause inconsistent thresholds for treatment in clinical practice. Advancing our understanding of postpartum hypertension is critical to strengthen preventive approaches and improve maternal cardiovascular health across the life course.

## Methods

This scoping review was conducted and reported in accordance with the Preferred Reporting Items for Systematic Reviews and Meta-Analyses extension for Scoping Reviews (PRISMA-ScR) guidelines^94^. The review followed a structured approach to identify, select, and synthesize published literature on persistent and de novo postpartum hypertension, focusing on pathophysiology, evaluation, and management. Study selection and data extraction were managed using Covidence (Covidence systematic review software, Veritas Health Innovation, Melbourne, Australia).

### Eligibility criteria

Eligibility criteria were developed using the Population, Concept, and Context (PCC) framework for scoping reviews^95^. Studies were eligible for inclusion if they focused on persistent or de novo hypertension in the postpartum period, defined as up to 12 months following delivery in accordance with the Centers for Disease Control (CDC) definition of pregnancy-relatedness^96^. Eligible studies were required to address at least one of the following domains: the pathophysiology, clinical evaluation, or management of postpartum hypertension. Only research articles published in the English language or translated into English were included. Studies were excluded if they were case reports or case series lacking generalizability or studies conducted exclusively in animal models. Study designs that were included comprised observational cohort and cross-sectional studies, randomized controlled trials, systematic reviews and meta-analyses, narrative reviews, implementation research, basic science studies, and relevant clinical practice guidelines and consensus statements. In addition, studies that focused solely on acute hypertensive emergencies without any follow-up data on sustained postpartum hypertension were excluded.

### Search strategy

A comprehensive search strategy was developed in collaboration with an experienced medical librarian. The search included both standardized vocabulary (e.g., MeSH and Emtree terms) and free-text keywords related to “postpartum hypertension,” “chronic hypertension,” “de novo hypertension,” and associated clinical terms. The initial search was conducted on March 15, 2024, across the following electronic databases: Ovid MEDLINE, Embase.com, and Scopus. The search was restricted to articles published between January 1, 2003, and the search date to reflect contemporary practice. A second search was performed on March 3, 2025, to capture newly published studies with the same criteria as before. Additional papers were included outside of the search criteria as long as they met the inclusion criteria as listed above.

### Study selection and data charting

Two reviewers (MG, AH) independently screened titles and abstracts for relevance. Full texts were retrieved for articles meeting the inclusion criteria or when eligibility was unclear. Disagreements were resolved through or in consultation with a third reviewer (ZM). Data were extracted using a standardized charting form designed for this study that captured key study characteristics, including author, year of publication, study location, and population. Additional variables included the type of postpartum hypertension (persistent or de novo), associated clinical features, management strategies, and reported outcomes. Extracted data were synthesized narratively to identify key themes and highlight gaps in the existing evidence.

### Evidence mapping

Included studies were categorized into four domains: epidemiology, pathophysiology, risk or protective factors, and management of persistent and de novo postpartum hypertension. Epidemiologic studies described the incidence, prevalence, and timing of postpartum hypertension across populations. Pathophysiologic studies examined mechanisms. Risk or protective factor studies evaluated maternal demographics, pregnancy-related conditions, and postpartum clinical predictors of sustained or newly diagnosed hypertension. Management studies focused on blood pressure monitoring strategies, antihypertensive treatment, follow-up timing, and models of postpartum care delivery.

## Data Availability

All data supporting the findings of this scoping review are derived from publicly available sources. The full search strategy is publicly available and can be accessed via the following 10.17605/OSF.IO/S3CVG.
